# Red Meat Amino Acids for Beginners: A Narrative Review

**DOI:** 10.3390/nu17060939

**Published:** 2025-03-07

**Authors:** Benjamin Barr, Danielle E. Levitt, Lauren Gollahon

**Affiliations:** 1 Department of Biological Sciences, Texas Tech University, 2500 Main Street, Lubbock, TX 79409, USA; benjamin.barr@ttu.edu; 2 Department of Kinesiology and Sports Management, Texas Tech University, 3204 Main Street, Lubbock, TX 79409, USA; danielle.levitt@ttu.edu

**Keywords:** red meat, protein, amino acid, essential amino acids, non-essential amino acids, semi-essential amino acids, overconsumption, metabolic-dysfunction-associated steatotic liver disease (MASLD)

## Abstract

Meat is a major source of dietary protein and fat across the globe. Red and white meat are the major terms consumers use to refer to types of meat; however, these terms do not fully encompass the range of nutrients provided by meat sources. Red meat refers to meat from mammalian skeletal muscle, while white meat refers to poultry. Red and white meat both provide a wide range of nutritional components in the context of fatty acids, amino acids and micronutrients. Importantly, it has been demonstrated that amino acid profiles differ between red meat and white meat as well as between different sources of red meat. Red meat is a complete source of dietary amino acids, meaning it contains all essential amino acids (EAAs), and in addition, it contains all the non-essential amino acids (NEAAs). Red meat is also the most abundant source of bioavailable heme-iron essential for muscle growth and cardiovascular health. Red meat has been indicated as a major contributor to the rising incidence of metabolic disorders and even colorectal cancer. However, it is important to note that while red meat consumption is linked to these conditions, it is typically the overconsumption of red meat that is associated with obesity and other metabolic symptoms. Similarly, the preparation of red meat is a key factor in its link to colorectal cancer as some methods of preparation produce carcinogens while others do not. Finally, red meat may also be situationally more beneficial to some groups than others, particularly in the cases of sex and aging. For pregnant women, increases in red meat consumption may be beneficial to increase the intake of semi-essential amino acids, while in the elderly, increases in red meat consumption may better preserve muscle mass compared with other dietary protein sources.

## 1. Introduction

Meat and meat products are a major component of diets worldwide. As a consistent dietary component, the underlying role of meat and meat products in the context of nutrition, disease, and aging is of particular interest. The focus of this narrative review is red meat, with specific emphasis on the role of amino acids in affecting metabolic-associated disease. This review also discusses the role of red meat proteins and amino acids with consideration to sex- and age-specific differences. Overall, this review aims to emphasize the importance of considering amino acid profiles of dietary components, especially in red meats. The search terms used in this review generally included the titles and subheadings with some variation of “red meat” or “red meat products”. In addition, as the focus transitions into the role of amino acids, search terms would include amino acid classifications such as “essential” or “non-essential”.

### 1.1. What Is Meat?

**Figure 1:**
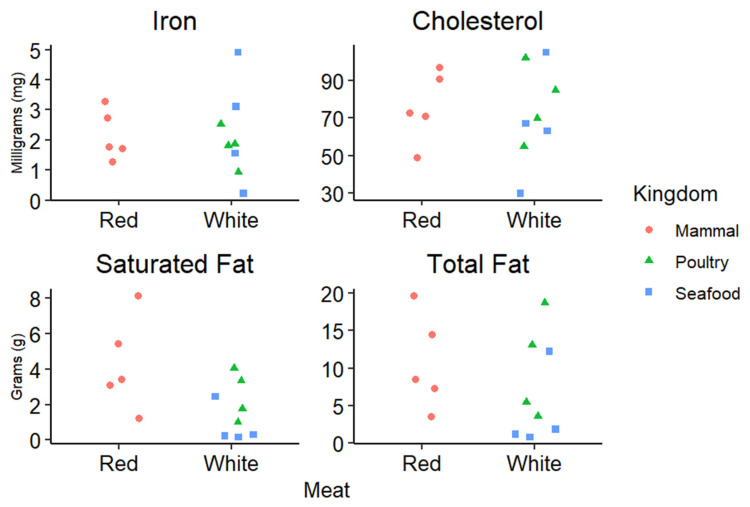
Concentrations of cholesterol, iron, saturated, and total fat measured in 100 g of meat protein, from 13 different types of meat. Meats surveyed were ground bison, beef, lamb, and turkey, flank steak, pork tenderloin, turkey leg, chicken nuggets, chicken breast, clams, shrimp, tuna, and salmon. Outliers were adjusted to fit on the graph for shrimp cholesterol (195 mg) and clam iron (26 mg). Original data from the USDA nutrient laboratory SR-21.

Meat and meat products are generally broken up into two categories, red and white meat [1,2]. Offal refers to meats other than skeletal muscle from either category (red or white), and similar to seafood, it is typically studied as its own category [1,2]. Red meat generally refers to any mammalian skeletal muscle such as beef, pork, and lamb, while white meat refers to skeletal muscle from poultry like turkey and chicken [1,2]. Interestingly, all poultry is considered white meat, even darker portions from leg muscle, simply because of its species of origin. Although these are the commonly accepted definitions of red and white meat, these terms are sometimes used interchangeably with meat containing various levels of unsaturated or saturated fat, which can cause confusion as to the nutritional composition of meats [1,2]. It is very apparent that these two terms are much too broad to accurately characterize the differences in the nutrient composition found in meat and meat products. To address this, the American Meat Science Association (AMSA) published the Meat Science Lexicon in 2018, which characterizes meat and meat products based on the type of processing and species [1]. Additional differences in red and white meat can be attributed to lipid profiles, mitochondrial densities, muscle fiber physiology, and even post-mortem handling of animals [2]. It has been suggested [2] that a more accurate terminology centered on a “specific set of parameters such as myoglobin, lipid profiles, or cholesterol content” would be more beneficial for both researchers and consumers. Figure 1 is adapted from Keeton and Dikeman [2], which describes a range of nutritional components and how they can vary within the categories of “red” and “white” meat.

### 1.2. Processed Meat

As indicated in Figure 1, while the AMSA has generated the Meat Science Lexicon for use in accurately characterizing meat, it does not break down meats based on nutritional components or values, but rather on the methods used for processing [1]. The major two categories are minimal and further processing [1]. It is important to note that meat and other products are processed for a variety of endpoints including preservation, storage, flavor, or protection from pathogens [1]. The minimal category lists raw meats that experience very little modification, such as fresh steaks or ground meats (with and without added ingredients) [1]. Examples of meat with minimal processing include marinated steaks and fresh sausages [1]. Further processing takes any minimally processed meat product and incorporates additional steps to increase shelf life, enrich flavor, or enhance convenience for consumers [1]. With the vast range of meat and meat products, it is crucial for researchers to use precise terms when discussing potential implications relating meat to dietary-based health outcomes. Based on this terminology, the remainder of the review will discuss health and nutrition implications of red meat and meat products with the goal of providing a concise and clear picture of current meat and meat products as nutrition sources. This review would also be remiss if it did not mention the IARC Monograph on Red Meat and Processed Meat [3]. Many publications before 2015 can be found in this Monograph, along with a thorough investigation into what was known about red meat and processed meat or meat products at that time.

## 2. Red Meat Composition

### 2.1. Fat

As briefly mentioned above, meat is a crucial source of dietary proteins worldwide, as well as a source of fats, vitamins and minerals. Fat in red meat is of great research interest. Saturated fatty acids (SFA) in dietary red meat has been associated with the development of obesity, cancer and cardiovascular disease [3,4]. Investigating the contributions of SFA in red meat led to the exploration other fatty acids, such as polyunsaturated fatty acids (PUFAs), omega-3 fatty acids (*n*-3) and trans-fatty acids in red meat [3,4]. Recently, data have shown that all SFAs may not contribute equally to the development of cardiovascular disease [5]. Differences were observed based on SFA chain length, where short and medium chain FAs were considered beneficial to neutral, while long-chain FAs were associated with increased cardiovascular risk [5,6]. These findings have continued to drive investigations into how modifications to fatty acid profiles in red meat can differentially impact health and improve dietary patterns [4,6,7]. In red meat, fat is subcutaneous, intramuscular, or intermuscular, and can vary based on the species, age, and sex of the animal [3,8]. In some cases, cuts of beef from older animals generally show increases in subcutaneous and muscular PUFAs, while the presence of SFAs may decline or remain the same [8]. Additionally, fatty acid profiles can be modified by altering animal feed [3,9]. When comparing grain-fed beef to grass-fed beef, grass-fed beef contained less SFAs and higher levels of PUFAs [9]. It has also been shown that the addition of flaxseed to a grain-based cattle diet can increase the presence of alpha-linolenic acid, a crucial *n*-3 [10]. Finally, and arguably the most relevant to consumers, are alterations to fatty acid profiles as the result of meat processing and cooking [3]. Cooking is one of the most critical steps in preparing red meat. With the high quantities of ready-made meals and products, this step can often occur before red meat reaches consumers, leading to questions regarding the fatty acid profile after cooking. However, in cooked pork and beef, changes to fatty acid profiles do not appear to be significant, meaning heat alone does not alter the type or quantity of FAs [11,12]. However, when red meat is cooked using additional fats, such as cooking oils or butter, the fatty acid profile of the red meat shifts toward those provided by the culinary fat [11,12]. For example, when pork was cooked with peanut oil (~17% SFA and ~56% MUFA) the quantity of SFAs in the meat decreased while MUFAs increased [11].

### 2.2. Vitamins and Minerals

Red meat is an important source of vitamin B_12_ and other B vitamins like thiamin or niacin [3,7,12]. They play key roles in many of the body’s cellular and physiological processes [7]. One of their most crucial roles is as coenzymes in the Krebs Cycle, activating enzymatically catalyzed redox reactions resulting in NADH or FADH_2_ to initiate the Electron Transport Chain and ultimately generate ATP [7,13]. Due, in part, to their roles in the Krebs Cycle, B vitamins are also key components in utilizing blood glucose and maintaining blood glucose homeostasis [7,13]. B vitamins are also utilized in the formation of heme proteins [13]. Additionally, red meat is a potential source for other vitamins like vitamin A and vitamin E; however, these are typically in lower concentrations than those of the B vitamins, especially vitamin B_12_ [7]. Interestingly, decreasing lipid quantity in red meat can increase the content of water-soluble vitamins (i.e., vitamin B), but does not appear to decrease the content of fat-soluble vitamins (e.g., vitamin A and vitamin E) [12]. This seems to occur when the fresh cut of red meat is leaner (i.e., the source animal was leaner while alive) and when some lipids are lost during red meat processing steps like cooking [12]. In uncooked steaks and in grain finished beef, those with higher fatty acid content had lower amounts of water-soluble nutrients [12,14]. After cooking, loss of some water-soluble nutrients is also observed; however, it is most likely due to moisture loss [12].

Red meat can also be a dietary source of essential minerals such as copper, zinc, selenium, and iron [7]. Interestingly, most mineral concentrations in red meat are not easily modified in livestock through dietary modification [7]. Thus, consumers need to appropriately modify their dietary intake based on their personal mineral needs and requirements [7,15]. Dietary consumption of any red meat (excluding offal) in the same quantity is likely to provide similar amounts of most minerals, except for iron [15]. This is largely due to the physiological roles played by each mineral. For example, copper is mainly regulated by and stored in the liver, so changes in red meat copper concentration are not likely to be observed from a nutritional standpoint [7]. Similarly, selenium levels are not likely to vary in red meat. However, this is due to the functionally essential role selenium plays in muscle contraction; any drastic changes in selenium concentration for muscle tissue result in muscular diseases effectively removing that animal as a dietary option [16]. Considering the wide ranges of iron depicted in Figure 1, findings from other sources agree that quantities of iron drastically vary not just from white meat to red meat, but under the category of red meat as well [2,7]. Heme iron is the portion of iron that is most bioavailable, with red meat being the most prominent dietary source [7,12,17]. For context, ~20–30% of heme iron is absorbed during digestion while only ~5–10% of non-heme iron is absorbed [7]. Although red meat is considered the premier source of dietary iron due to its heme component, overconsumption can lead to heme- and iron-based gastrointestinal complications such as colorectal cancer [7,18]. These complications are discussed further in the context of proteins, amino acids and health.

### 2.3. Protein and Amino Acids

Since the major focus of this review is protein in red meat, protein as a macronutrient is discussed in a brief, but in-depth overview. Meat is an important source of protein, and thus, an important source of amino acids (AA). Although technically dietary protein is the major consideration for most consumers, protein is nutritionally inert unless first broken down by proteases and peptidases into AAs (or short chain peptides) [19]. The chemical digestion of dietary protein begins in the stomach with chemical denaturation of protein due to low pH (0.8–3.5) and further cleavage of the denatured AA chains by pepsin [20]. This results in the partial digestion of protein and further breakdown occurs in the small intestine with the aid of a range of pancreatic proteases (e.g., trypsin, chymotrypsin, carboxypeptidase, and elastase) [20]. At this point, the usable components of dietary protein have been broken down into one of the 20 L-isomer, α-AAs (or di- and tripeptides) absorbable by the cells of the small intestine [21]. Meat is considered a complete dietary protein, meaning it contains all of the essential amino acids (EAA) [22]. Additionally, meat contains 11 non-essential (NEAA) or semi-essential amino acids used by the human body [22]. As previously mentioned, AAs are commonly divided into 3 categories in the context of nutritional requirements: EAA, NEAA and semi-essential amino acids [21,23,24]. EAAs, also known as indispensable amino acids, are those which the body is unable to produce in sufficient quantities internally. NEAAs, also known as dispensable amino acids, are those the body is naturally able to synthesize in quantities adequate for survival [21,24]. The authors would like to note at this point the terms “indispensable” and “essential” may be used interchangeably and the same is true with “non-essential” and “dispensable”; in order maintain consistency this paper will continue to use “essential” and “non-essential” except when necessitated by official names like with Digestible Indispensable Amino Acid Score (DIAAS) described below.

The 20 AAs utilized by humans for biological functions are listed in Table 1. Semi-essential amino acids are those which are essential, or insufficiently produced, under certain stress conditions such as disease states, or life stages like early childhood or pregnancy [21,24]. Based on the wide range of possible stress conditions, there is currently disagreement on whether all NEAAs should be considered semi-essential, with some authors arguing for the “dietary essentiality of ‘nutritionally non-essential AAs’ for humans” [24]. While one of the major uses for AAs is in protein synthesis, AAs also play key roles in biological functions such as maintenance of acid-base balance, hormone secretion, and nutrient metabolism [21,24]. Additionally, as the main source of exogenous nitrogen, AAs play a crucial role in maintaining nitrogen balance within the body [24].

AA digestibility is assessed using true fecal digestibility or less commonly, true ileal digestibility [25,26]. Studies in understanding the gut microbiome and nutrient absorption have indicated that this simple method of measuring AA absorbance can be insightful but may have complications [25,26]. Fecal AA profiles contain both residual dietary AAs and endogenous AAs [25,26]. Endogenous AAs are produced by the gut microbiome as well as those from intestinal mucosa and digestive enzymes [25,26]. True fecal digestibility corrects for the presence of endogenous AAs and more accurately reflects absorption of dietary AAs [26]. A more optimal method to measure dietary protein availability is to measure ileal digestibility, or the ratio of AAs present at the terminal end of the ileum after consumption of the dietary protein source (DPS) [25,26]. Similar to true fecal digestibility, endogenous AAs are corrected for when calculating true ileal digestibility, albeit without the need to consider contributions from the large intestinal microbiome [25]. However, due to invasiveness of ileostomies, this method is not as commonly used as assessing fecal digestibility [27]. Utilizing true fecal digestibility and true ileal digestibility, the Food and Agriculture Organizations of the United Nations (FAO) generated two methods to assess protein quality in dietary sources. Developed in 1991 and using fecal digestibility, the Protein Digestibility Corrected Amino Acid Score (PDCAAS) is calculated using the amount of each EAA in a DPS of interest relative to a reference DPS to generate an individual “amino acid score” [28,29]. The lowest “amino acid score” is then normalized by the average true digestibility of the DPS for the PDCAAS [29]. PDCAAS had major gaps including heavily relying on fecal digestibility and a focus on only the first limiting EAA in each source [28]. Then, in 2011, the FAO released an updated method to assess protein availability using ileal digestibility, the DIAAS [28]. The DIAAS uses individual EAA ileal digestibility to generate a score for all present EAAs and then bases the complete dietary protein source’s DIAAS on the lowest AA’s digestibility score [29]. Therefore, DIAAS can be used to provide a more robust and complete assessment of what EAAs are provided by a DPS than was previously available using PDCAAS [29]. Although DIAAS does provide a more robust assessment, a few limitations remain. Due to the invasiveness of ileostomies, the number of DPS which have been assessed using DIAAS is limited [29]. Additionally, DIAAS would typically only consider EAAs and not NEAAs or semi-essential AAs which may be crucial in some disease states [29].

Red meat, or meat from mammalian skeletal muscle, it is a major dietary protein source, and by consequence, an AA source [30]. Depending on the species (and other individual factors related to the animal), 100 g of cooked red meat contains ~28–36 grams of protein [30]. For example, 100 g of cooked white meat contains ~23–31 grams of protein, while 100 g of vegetables only have around 3–8 grams of protein [31,32]. Additionally, it has been found that these different dietary protein sources (DPS) also have different digestibility due to their AA composition [26]. A WHO report, adapted by Moughan and Wolfe (2019), indicates that meats (red, white and fish), and animal products demonstrate a true fecal digestibility of ≥94% [26]. In contrast, plant dietary protein source fecal digestibility ranges from 72–99% with a majority being less than 90% [26]. Compositionally, most dietary protein sources considered red meat have very similar AA profiles; this is the result of myofibrillar proteins making up ~70% of the total skeletal muscle protein content [33]. However, the quantities of individual AAs may be different due to variations in functional requirements of skeletal muscle and intramuscular fat profiles between species [33,34]. In a comparison of AA variability in the human diet, Dai et al. demonstrate the wide range of AA quantities across meat (and plant based) DPS [35]. Similarly, in the assessment by Kaczmarska et al. using LC-MS, beef and pork had up to 32% differences in EAA proportions and more than 100% differences in NEAA proportions as demonstrated in Table 2 [36]. Interestingly, in the context of ground meat products Fanelli et al. demonstrated that quantities of digestible AAs in 93% lean ground beef burgers more closely resembled 80% lean pork ground burgers than 80% lean ground beef burgers [37]. Supporting these findings, differences in EAAs and NEAAs can be found in different cuts of meat from the same animal [38]. In 2016, Wu et al. examined the AA quantities in three different cuts of beef, chuck, round or loin, from the same 10 animals [38]. They determined that almost every AA examined (EAA and NEAA) was significantly different in total quantity for at least one of the cuts when compared with the others [38]. In most cases, chuck roasts were those markedly different with round and loin being more similar [38]. Further, in the same species and cut from the same location, red meat can demonstrate different AA content based on sex and age [3]. In 2018, a study by Hodgkinson et al. showed that different methods of cooking red meat may slightly alter the quantity of AAs; however, it does not significantly alter the DIAAS [39]. Variation in AA profiles of red meat can be found as the result of species, age, sex and even cuts of meat within a species. AAs can also form AA derivatives like taurine or glutathione. To the authors’ knowledge, it remains unknown how AA profiles of DPS influence the profile of AA derivatives other than predictable relationships between precursors and their downstream derivatives. Additionally, it is unclear how various processing methods affect AA derivative profiles. The specific roles of each AA in health and disease have been explored in an individual context in a recent review by Ling et al. [40]. The review by Ling et al. provides context for the molecular roles of AAs in a range of diseases including cancer, but from a more molecularly dedicated approach [40]. Additionally, the mentioned review discusses evidence for a range diet formulations either supplementing or eliminating amino acid components in preclinical models [40]. Although a powerful article, it does not examine the components of dietary protein sources nor the contributions of dietary patterns to systemic AA profile [40].

AA derivatives like taurine, carnitine, creatine, carnosine, and glutathione are critical nutritional components [38,41]. Similar to semi-essential AAs, these bioactive AA derivatives can be synthesized endogenously within the body; however, in times of stress, and especially during physical activity, endogenous production is not sufficient [42]. Taurine and carnitine play key roles in anti-oxidative and anti-inflammatory pathways [42]. Carnitine also helps transport fatty acids into the mitochondria where they undergo β-oxidation to produce energy when glycogen stores are low [42,43]. Creatine regulates cellular ATP and has antioxidant properties [42,44]. Carnosine also acts as an antioxidant and anti-inflammatory agent while playing a role in pH-buffering and in reducing lipid peroxidation [42,45]. Finally, glutathione acts as a master antioxidant, regulating free radicals within cells while also detoxifying cells, among other roles [42,46]. Although the targeted AA derivative enrichment of a DPS remains unexplored, it does appear that post-mortem processing of a DPS may influence AA derivative profiles [47]. Tangentially, AA derivatives, particularly carnitine and taurine, are added to beverages, specifically energy drinks for their stimulating capabilities related to muscle function [48]. Although AA derivatives in beverages are outside the scope of the current review, the authors would like to highlight that intake of AA derivatives in liquids rather than as part of a solid dietary component should alter their absorption speed and likely their functional profiles since they are able to more rapidly progress through the digestive tract [49].

Previously, information concerning EAA, NEAA and semi-essential amino acid profiles along with AA derivatives, some of which were discussed above, was mostly emphasized in the fields of sports nutrition and muscle science. However, recent interest in “Food as Medicine” and nutraceuticals is elucidating gaps which could potentially be addressed by AA and AA derivative dense sources.

## 3. Discussions

### 3.1. Amino Acids in Sports Nutrition

The application of nutritional science in conjunction with exercise science has been one of the most crucial avenues for driving investigations in food science research [42]. As such, the optimal nutritional requirements necessary for improving and maintaining athletic performance continue to be thoroughly investigated [42]. As discussed above, red meat provides both fatty acids and protein, two nutrients crucial to physical activity [42]. Fatty acids provide energy in muscle cells through β-oxidation to preserve glycogen stores and as glycogen stores become depleted [43]. Proteins, and thus AAs, are critical for muscle growth, recovery, and maintenance [42]. As stated above, meat is a complete protein, making it a source for any AA crucial for muscle repair. For example, glutamine can increase glycogen synthesis in muscle cells after exercise, improving tissue repair [50]. Glutamine also improves the tightly regulated inflammatory response in skeletal muscle during recovery from exercise [50]. Additionally, as a precursor to glutathione, increases in glutamine also increase the clearance of reactive oxygen species following tissue damage [50,51]. Included in the EAAs found in red meat are the branched chain amino acids (BCAAs), valine, leucine and isoleucine, with leucine at particularly high concentrations [36,52]. BCAAs are crucial in the protein synthesis required for muscle repair, and a complete protein source is the most optimal for repair [52]. Notably, supplements containing only BCAAs are nearly as effective in maintaining protein synthesis as complete protein sources [52]. Leucine in particular has been shown to stimulate muscle protein synthesis [52]. Indeed, it has been demonstrated that leucine can directly activate signaling through the mammalian target of rapamycin (mTOR) pathway, which promotes insulin-dependent pathways related to muscle repair [52].

### 3.2. Cardiovascular Disease

Red meat continues to be associated with the development of cardiovascular disease; however, due to the complex nature of co-morbidities like obesity and type 2 diabetes, their link remains unclear [53]. One potential link is through trimethylamine-N-oxide (TMAO), a metabolite generated from carnitine and choline by the gut microbiota [54]. In addition to TMAO synthesis by the gut microbiota, TMAO can be directly absorbed in the small intestine. Interestingly, the highest concentrations of TMAO in dietary meats are found in fish and marine invertebrates [54]. When consuming 6 oz of fish compared with 6 oz of beef, serum TMAO concentrations were 50 times higher in individuals consuming fish [54,55]. Serum TMAO concentrations are typically altered through modification of DPS intake [54]. Although further investigation is still required, metabolism of carnitine into TMAO from red meat could be a major finding in the characterization of dietary related disease [54]. As previously stated, carnitine plays a crucial role in β-oxidation by shuttling fatty acids in to the mitochondria [55]. It is possible that a substantial portion of carnitine from red meat is converted to TMAO, leaving less to facilitate the movement of fatty acids into mitochondria. If this is the case, it could be a potential reason for the development of metabolically associated disease comorbidities. Besides TMAO, disentangling red meat from the comorbidities above continues to perplex investigators but further investigations into lipid profiles of red meat sources are promising [53]. Dietary modification of AAs, and particularly BCAAs, offers potential new routes for treatment of cardiovascular disease; however, most AA associations with cardiovascular disease are as markers of disease rather than causes [56].

### 3.3. Red Meat, Obesity and Type 2 Diabetes

The relationships between red meat, meat consumption and obesity are complex and multifaceted. The narrative over the past years has been that red (and processed) meat consumption is associated with increased levels of obesity [57]. However, study findings typically report that it is not red meat consumption but overconsumption that tends to lead to high incidences of obesity [58,59]. In fact, recent trending dietary patterns for weight loss include the carnivore diet, a meat-only diet, which claims reductions in total body and fat mass while maintaining fat-free or lean mass [60]. Despite implications for weight loss, there is only anecdotal evidence supporting the use of a meat-only diet for long-term health improvement. In addition, the relationship between type 2 diabetes and red meat or protein intake remains unclear. Due to its association with obesity, it makes sense that the overconsumption of meat, particularly red and processed meat, would increase incidence of type 2 diabetes. Studies have shown that high consumption of red meat is positively associated with the development of type 2 diabetes [61]. Again, it is prudent to note that these studies rarely consider types of red meat and rely heavily on self-reporting without a metric for physical activity [58,59,60,61]. Criteria for self-reporting also varies; in some cases, it is 24-hour dietary recall, while in others, it is long term approximation of consumption of a specific dietary component [58,59,60,61]. Additionally, as demonstrated in a 2020 study of patients on their initial visit to a Diabetology center, major limitations in self-reporting include inability to complete nutritional questionnaires independently [62]. This, along with other exclusion criteria, resulted in a relatively small number (~76) of 390 patients able to complete the study [62]. Although a majority of those assessed scored higher than “sufficient” in terms of nutritional knowledge, almost 1/3 of participants evaluated to be “insufficient” [62]. Lack of nutritional education and access to nutritional education providers often results in poor health outcomes; however, the approach used by nutritional education providers is also just as important to consider [63,64]. Nutritional literacy has been linked to healthier food consumption, which is a major component to preventing the onset of obesity [65].

A key component in overconsumption, especially in the cases of obesity and diabetes, is satiety signaling [66,67]. As indicated above, red meat is a complete protein source, and dietary protein plays a strong role in satiety signaling, which is a contributing factor to the weight loss observed in high protein diets [68]. This is due in part to increased AA concentration in the blood and increases in satiety-related hormones like GLP-1s, and CCK [68]. Impaired leptin signaling is also a contributing factor to the development of obesity and, in turn, type 2 diabetes. The consumption of red meat has been demonstrated to differentially affect leptin levels based on fat content and processing of meat [66,67]. In most cases, lower fat content in a DPS results in higher gastric leptin secretion, as a portion would contain more protein [69]. It has been demonstrated that high-protein (and thus high AA content) diets cause increased secretion of gastric leptin after consumption compared with high fat diets, indicating high-protein diets provide more satiety signaling than high fat diets [69]. Typically, chronically high consumption of both red meat and processed meat results in increases in serum leptin due to elevated amounts of fat mass in consumers [66,67]. Additionally, it appears that serum leptin is similarly affected by red meat consumption in both sexes, although it is likely that this is confounded by high fat mass in consumers [66]. There is little to no information available on differential leptin expression from varying red meat sources and so it remains unclear if changes to leptin signaling are the same regardless of DPS. Furthermore, complications from the onset of type 2 diabetes can often be linked to elevated levels of specific AAs or may be resolved by supplementation with other AAs or their derivatives [70,71,72]. Such complications include diabetic retinopathy, neuropathy and nephropathy [70,71,72]. In a metabolomic assessment of various retinopathies, elevated serum glutamine was linked to each and especially emphasized in diabetic retinopathy [70]. In contrast, it has been suggested that supplementation with amino acids, in particular the AA serine, may prevent or delay the onset of diabetic neuropathy [71]. Finally, AAs can also display optimal levels of function, for example glutamine in diabetic kidney disease [72]. Appropriate levels of glutamine have been shown to prevent oxidative stress and promote glucose homeostasis, but excessive supplementation has been demonstrated to increase biomarkers of inflammation [72]. This nuance in AA levels and disease emphasizes the importance of exploring the AA profiles in DPS, to contextualize nutritional sources for maintenance of health and prevention of disease. In the context of red meat consumption, it is again probable that AA metabolism is disrupted by over consumption resulting in excessive levels of most AAs.

### 3.4. The Gut Microbiome

The gut microbiome is heavily influenced by dietary composition and has been identified as a major contributor to overall health and the onset of disease [73,74]. Typically, when included in the diet, red meat supports the diversity of the overall microbiome [73,74]. Although it has been demonstrated that red meat intake can alter the gut microbiome, it remains unclear whether or not these alterations are strictly beneficial or detrimental [73,74]. For example, current knowledge indicates that there appears to be an optimal dietary intake of digestible dietary protein for promoting a healthy gut microbiome, levels of which are rapidly met by red meat DPS [74]. It is likely that the high digestibility of AAs from red meat restricts their ability to directly support AA auxotrophic bacteria populations within the large intestine [75]. However, it is also possible that, from host–symbiote cross feeding, higher levels of serum AAs and, in turn, AA availability in the host supply AA auxotrophies with the necessary AAs they require [75]. Additionally, as mentioned in the discussion of digestibility, excess AAs from red meat not absorbed by the small intestine may be further altered in the large intestine by the microbiome, which can still be absorbed depending on bacterial modifications [76,77]. Ultimately, although certainly crucial to the overall health of an individual, the impact of AA profiles in red meat, and in fact AA profile in general require further exploration in the context of the gut microbiome [77]. At present, this exploration is taking place by identifying the functional roles of particular genus and species within the gut microbiome. Further exploration in the context of AAs and the microbiome will be required to improve our understandings of the nature of complex host-microbe and host–microbial community interactions as well as interplay within microbial communities found in the gut [77].

### 3.5. Colorectal Cancer

In the 2015 IARC Monograph, the working group determined that “red meat is probably carcinogenic to humans”, and this is still reported as correct from other sources in 2021 [3,17,78]. Carcinogens in red meat have most strongly been linked to incidence of colorectal cancer (CRC) [3,78]. Carcinogenicity in red meat is due to the formation of heterocyclic aromatic amines (HAAs) and polycyclic aromatic hydrocarbons (PAHs) [3]. Both HAAs and PAHs are formed during processing or cooking, and high-temperature cooking with direct contact to the heat source (e.g., grilling, smoking, or pan-frying) seems to be the main culprits in HAA and PAH generation [3]. HAAs are formed when free AAs interact with creatine under high heat conditions [3,79]. Further research has expanded on red meat carcinogenesis and a systematic review reports more links between specific HAAs, PAHs and excessive quantities of heme iron to provide stronger evidence for initiation of CRC [78]. Obesity is also considered a risk factor for colorectal cancer, meaning overconsumption, type 2 diabetes and dysregulated leptin signaling could all play key roles in the development of CRC. While the presence of the carcinogens in red meat discussed above do contribute to development of CRC, further investigation is required to identify if they cause cancer in other tissues [78]. Once again, there is very little information on differences in CRC incidence based on individual red meat sources. However, depending on the concentrations of pre-carcinogens, it is possible that any red meat processed or cooked in a particular manner could contain sufficient carcinogens to induce CRC or other types of cancer.

### 3.6. Metabolic-Disorder Associated Steatotic Liver Disease (MASLD)

In 2023, the term metabolic-dysfunction-associated steatotic liver disease (MASLD) was proposed to replace metabolic associated fatty liver disease (MAFLD), formerly non-alcoholic fatty liver disease (NAFLD, 2020), and is the most common liver disease globally [80,81]. MASLD more accurately describes the development of fatty liver disease in the presence of metabolic dysfunction associated with obesity and other metabolic disorders [80]. Studies have strongly associated red meat consumption and the development of MASLD across the globe [82,83,84]. Almost all studies investigating a link between red meat consumption and MASLD are heavily reliant on individuals who have obesity [82]. In December 2024, Liao et al. identified dietary protein as the key contributor to the development of de novo lipogenesis (DNL) in the liver in obese mice [85]. Specifically, this study demonstrated that AAs, especially EAAs, are utilized as the carbon backbone for ~75% of DNL in the liver [85]. These findings were based on previous trends linking MASLD to medium to high protein (>33% total kcal from protein) intake in obese, but not normal or overweight, individuals included in the National Health and Nutrition Examination Survey dataset [85]. Notably, a common trend among obesity-dependent diseases, particularly in the case of MASLD, is that those who are more obese tend to consume, or rather overconsume more red meat [82,83]. Indeed, one such study to link red meat to MASLD indicated just such a finding, that there appeared to be a dose-dependent relationship between consumption of red meat and MASLD development [86]. There have been notable findings relating nutritional composition to incidence of liver disease. One study investigated non-obese patients with fatty liver disease (what would now be considered MASLD), and found that modulation of dietary cholesterol may prove to alter the development of MASLD [87]. Another study focused on animal protein sources and how they may differentially contribute to MASLD [88]. Here, the authors suspect a link between red meat and MASLD via the capacity of the liver to store iron [88]. The liver acts as the main store of iron within the body and as such impairments in liver function cause increased accumulation of iron within the liver [88,89]. In the case of comparing animal protein sources it is postulated, but not established, that difference in MASLD incidence may be due to high bioavailability of heme iron in overconsumption of dietary red meat [88].

### 3.7. Red Meat and Biological Aging

Aging is a broad and complex topic, generally including the onset of degenerative disease, as well as cellular signs of aging such as telomere shortening, although ultimately, the true measure of aging is a declining quality of life. Other metrics of aging include impaired cognitive ability and increased frailty. Additionally maintaining a healthy dietary pattern is crucial to delaying the progression of biological aging, as it reduces outcomes associated with early initiation of the aging process [90]. In the context of amino acids, various combinations of restrictions and supplementations have been found to be both pro-and anti-aging in pre-clinical models [91]. Although a range of combinations have been examined, restrictions in methionine or tryptophan appear to be essential in delaying aging in pre-clinical models [92]. Currently, red meat is not among the constituents highly recommended to promote healthy aging, in part due to the propensity for it to be consumed in high levels as demonstrated by the studies above including cardiovascular disease, obesity, cancer, and MASLD [61,78,82,83,90]. Additionally, high red meat consumption has been linked to elevated levels of reactive oxygen species, which may inhibit telomerase or increase telomere shortening, primarily in enterocytes [93,94]. Telomerase inhibition in enterocytes has yet to be demonstrated as the result of red meat consumption, but it has been demonstrated in the consumption of processed meat [94]. Long-term red meat consumption has also been linked to an earlier decline in global cognition and verbal memory [95]. Further, higher rates of red meat consumption was also associated with increased incidence of dementia in older individuals [95]. Although linked to a decline in neurological function, the availability of AAs in red meat can also provide some structural benefits. For example, increasing protein consumption can be an incredibly beneficial dietary modification in aging individuals suffering from sarcopenia or frailty [96]. Typically, studies in aging focus on the late life impacts or outcomes after the age of 60 [96,97]. It was indicated that middle aged cohorts (50–65) consuming high-protein diets had higher all-cause mortality than those consuming low protein diets [96]. However, in the cohort above age 66, a high-protein diet was consistent with lower all-cause mortality [96]. This provides strong evidence for dietary modification based on need and life stage. The above issues with HAAs, PAHs and overconsumption, in addition to high fat content of choice red meat cuts, makes it difficult to recommend the use of red meat as the sole protein source for aging individuals [96,97]. However, red meat as part of a complete diet appears to be the most optimal in extending the longevity and quality of life, demonstrating lower all-cause mortalities and improvements in lean mass for elderly individuals [96,98]. Additionally, modifications to the composition of red meat during processing or utilization of leaner cuts of meat provide potential avenues to take full advantage of AA profiles present in red meat [96]. Furthermore, studies have identified targets for dietary intervention like monitoring intake of specific AAs, like methionine, or excessive BCAAs in elderly individuals who are obese [96]. Finally, it has been identified that serum AA levels most likely reflect habitual dietary habits, or dietary patterns established over a prolonged period of time, which may be crucially important for identifying *when* to transition to a higher protein diet [99].

### 3.8. Red Meat and Sex

Scientific and particularly biomedical research has made many strides over the years relating medical findings to the population as a whole. In 2016, the NIH instituted a policy that requires any NIH funded research to include sex as a biological variable (SABV), and since then, incredible strides have been made in clinical and preclinical trials considering SABV [100]. Studies in nutrient consumption have identified different dietary patterns in males versus females post puberty along with characterizations of sex-based disparities of disease incidence [101,102,103]. Additionally, when incorporating excess red meat into the diet, men exhibit different metabolic biomarkers than women [104]. For example, high red meat consumption in men produces excesses in serum creatinine more rapidly than those found in women [104]. This is, in part, due to the higher quantities of muscle mass present in males when compared to females [104,105]. In the same study, BCAAs were elevated in females in association with red meat consumption when compared with males, potentially indicating fewer BCAAs were utilized for protein synthesis [104]. In contrast, another study including a healthy cohort for comparison, identified that serum BCAA was decreased in females compared with males, and healthy females had no different BCAA levels than obese females [106]. Additionally, both studies identified high variance in lipid containing metabolites like cholesterol, HDL, and LDL between men and women consuming red meat; suggesting alternative yet unestablished metabolic differences between sexes likely linked to sex-dependent requirements for hormone synthesis and signaling [104,106]. Notably, other disease states relating to red meat consumption are also observed to have sex disparities. Most recently established is the disparity observed in MASLD. Globally, it is estimated that 30–40% of men and 15–20% of women are affected by MASLD [107]. As mentioned above, red meat is a major contributor to serum BCAAs even if the exact method of their contribution between sexes is yet to be fully understood. It was found in a study cohort that MASLD severity was positively associated with plasma BCAAs, particularly in women [108]. In fact, when examining the progression of MASLD into fibrosis, BCAAs were only found to markedly increase in women [108]. Although findings relating alterations in fat metabolism seem to match with understanding the nuances of protein metabolism, further investigation is required in the context of both red meat and sex disparities.

### 3.9. The Future of Red Meat

Interestingly, recent findings indicate that skeletal muscle, apart from its role in movement, also plays a role in systemic endocrine signaling which can influence health and disease in a sex-dependent manner [109,110]. As the role of skeletal muscle in systemic signaling is uncovered, it will be important to keep in mind a wide range of dietary protein sources, including red meat, with the potential to support its proper function. Furthermore, recent pre-clinical studies have investigated modification of dietary protein sources using treatments during processing, such as ammonium hydroxide enhancement (AHE), to potentially improve taste and metabolically associated health outcomes as well [111,112]. The studies found improved metrics of metabolic health from beef protein sources treated with AHE, including improvements to glucose clearance in C57Bl/6 male mice and increased longevity and decreased incidence of cancer and MASLD in C3H/HeJ mice of both sexes [111,112]. Additional avenues of processing for red meat and red meat products should include attempts to optimize AA profiles, either through finishing diets for the DPS or through alterations utilizing direct supplementation of AAs incorporated into the product. Aspects of research centered around red meat should include full characterization of its association with improving physical health as well as its contributions to metabolic disease. Finally, continued exploration of consumption compared with overconsumption of dietary protein, particularly in the context of red meat is crucial in determining how red meat fits into a healthy dietary pattern.

## 4. Limitations and Strengths

The current review provides an overview of the metabolic and nutritional value of amino acids in the context of red meat. The review is limited in scope to focus only on red meat and does not attempt to provide an in-depth exploration of amino acids from DPS outside of that category. Additionally, based on the scope of the review, it only provides a cursory assessment of amino acid derivatives, like taurine or carnosine. This review provides a bridge between the molecular duties of amino acids and a specific DPS. By focusing on AAs in red meat, this review was able to identify nuances in a category of DPS broadly used by consumers. It was also able to provide a brief overview of tools to assess amino acid components like PDCAAS and DIAAS.

## 5. Conclusions

Red meat is a complete source of dietary protein and can include a reasonable amount of important dietary fats. The overconsumption of red meat has been associated with the development of metabolic related disorders in both sexes, but the exact mechanisms as to how this occurs in most cases have yet to be determined. Red meat provides the highest quantities nutrients essential for skeletal muscle growth, and in some cases improves quality of life in older individuals. Due to the controversies surrounding red meat, further investigation is required to determine how it can be best incorporated as a part of a healthy diet.

## Figures and Tables

**Table 1:** Complete list of essential and non-essential amino acids obtained from dietary sources.

Essential	Non-Essential
Histidine *	Alanine
Isoleucine	Arginine *
Leucine	Asparagine
Lysine	Aspartic acid
Methionine	Cysteine *
Phenylalanine	Glutamic acid
Threonine	Glutamine *
Tryptophan	Glycine
Valine	Proline
	Serine
	Tyrosine *

Known semi-essential amino acids are indicated with “*”. A “*” has also been given to NEAAs labeled as essential in other sources [21,23,24].

**Table 2:** Percent difference of non-volatile AAs found in red meat (beef and pork), determined by liquid chromatography-mass spectrometry analysis adapted from Kaczmarska et al. [36].

EAA				NEAA			
AA	Beef	Pork	% Difference	AA	Beef	Pork	% Difference
Histidine *	129	93	32.43243	Aspartic acid	5	18	113.0435
Leucine	1059	799	27.98708	Tyrosine *	15	49	106.25
Isoleucine	560	453	21.12537	Glutamine *	1375	674	68.42362
Phenylalanine	491	400	20.42649	Asparagine	28	21	28.57143
Methionine	251	296	16.45338	Serine	98	76	25.28736
Lysine	366	311	16.24815	Proline	966	1148	17.21854
Tryptophan	38	38	0	Glutamic acid	405	460	12.71676
				Alanine	319	334	4.594181
				Glycine	25	24	4.081633

“*” for AAs indicate semi-essential (see Table 1).
